# Roles and Clinical Applications of Biomarkers in Cardiovascular Disease

**DOI:** 10.1155/2016/8982796

**Published:** 2016-07-05

**Authors:** Stefano de Franciscis, Laurent Metzinger, Raffaele Serra

**Affiliations:** ^1^Interuniversity Center of Phlebolymphology (CIFL), International Research and Educational Program in Clinical and Experimental Biotechnology, University Magna Graecia of Catanzaro, Viale Europa, 88100 Catanzaro, Italy; ^2^Department of Medical and Surgical Sciences, University Magna Graecia of Catanzaro, Viale Europa, 88100 Catanzaro, Italy; ^3^CURS, Laboratoire INSERM U1088, Université de Picardie Jules Verne, Chemin du Thil, 80025 Amiens Cedex 1, France

Due to the high rates of mortality and morbidity caused by Cardiovascular and Peripheral Vascular Disease (CPVD), great efforts have been made in order to improve diagnosis and primary and secondary prevention and treatment, even by discovering and using related biomarkers. As such, the articles contained in the present issue include both reviews and research articles focused on characterizing several biomarkers in order to evaluate their role in the field of CPVD.

The review by S. de Franciscis et al. considers the current evidences of novel biomarkers with clear implications in the risk assessment, prevention strategies, and medical decision making in the main fields of CPVD, such as Hypertension, Coronary Heart Disease, Arterial Aneurysms, Carotid Artery Disease, Peripheral Artery Disease, Chronic Venous Disease, and Venous Thromboembolism.

The study by Y. Hao et al. shows multiple metabolites that associated with risk of developing Hypertension (HYT). Specifically, low amino acid levels and gut microbiome seem to play an important role in the pathogenesis of this disease.

J. Xu et al. confirm the already known association of genetic polymorphisms of ATP2B1 with the susceptibility to HYT in the Han Chinese population, particularly in the females, and found that the interaction of high BMI and ATP2B1 variants increased even more the susceptibility to Hypertension.

Acute heart failure (AHF) is the most common reason of hospitalization in patients aged 65 and older, with mortality rate up to 30–40% within one year, and, in this context, Y. Wang et al. show that sequential monitoring of changes of N-terminal probrain natriuretic peptide (NT-proBNP) within the first days after acute heart failure (AHF) may be helpful for guiding clinical management of AHF patients.

In ST-elevation myocardial infarction (STEMI) patients, it is pivotal to prevent AHF and recurrent thrombosis, and, in this context, M. Marinšek and A. Sinkovič show the beneficial effects of Ramipril and Losartan associated with dual antiplatelet therapy (DAPT) in respect to DAPT alone, by means of measuring the level of several biomarkers such as NT-proBNP, ejection fraction (EF), plasminogen-activator-inhibitor type 1 (PAI-1), and platelet aggregation by closure times (CT).

As atherosclerosis and vascular calcification are dynamic processes, T.-L. Chuang et al. studied the association between the bony microarchitecture score (trabecular bone score, TBS) and coronary artery calcification (CAC) in adult subjects undergoing health exams and they found that advanced age was significantly associated with high CAC, while increased TBS was associated with moderate CAC, independent of age and other risk factors, and they concluded that further evidences were needed to confirm their data.

Osteoprotegerin (OPG) and its ligands, receptor activator of nuclear factor *κ*B ligand (RANKL) and TNF-related apoptosis-inducing ligand (TRAIL), are known players in osteoblastogenesis and osteoclastogenesis. In fact, they now represent also new biomarkers in the atherosclerosis and vascular calcification fields, as described by S. Bernardi et al. with evidences pointing towards a proatherogenic role for OPG and an antiatherogenic role for TRAIL.

Misdiagnosis in Pulmonary Embolism (PE) may increase the related high mortality, and E. Gul et al. investigated the role of serum adiponectin levels and they found that adiponectin levels were significantly low in the patient group with PE compared to patients without PE, and they concluded that these findings may have important implications in the diagnosis of PE.

The current literature abundantly describes that microRNAs (miRNAs) and long noncoding RNAs are potential new biomarkers for most pathophysiological processes, including cardiovascular diseases and metabolic disorders. In this special issue, L. Louvet et al. show, using a human* in vitro* model of vascular smooth muscle cells, that several miRNAs are implicated in the preventive role of magnesium towards vascular calcifications. A. Carino et al. show that the levels of several circulating miRNAs are altered during the switch from clopidogrel to ticagrelor. Their results hint at the possibility of using miRNAs as noninvading biomarkers to determine primary end points in the CPVD field, although further work will be necessary to clearly establish that. Finally, the very recent discovery of long noncoding RNAs, defined as non-protein coding transcripts longer than 200 nucleotides, opens new horizons in the CPVD field, as evidenced by the paper of Y. Yan et al., that shows that the circulating level of the long noncoding RNA UCA1 is altered during AHF.

We hope that this special issue would throw light on the major issues in the area of biomarkers in CPVD and would attract the interest of scientific community in order to pursue further investigations leading to the discovery of novel biomarkers and to their rapid implementation into clinical practice.

*Stefano de Franciscis* *Laurent Metzinger* *Raffaele Serra*

